# Accuracy of Continuous Glucose Monitoring before, during, and after Aerobic and Anaerobic Exercise in Patients with Type 1 Diabetes Mellitus

**DOI:** 10.3390/bios8010022

**Published:** 2018-03-09

**Authors:** Lyvia Biagi, Arthur Bertachi, Carmen Quirós, Marga Giménez, Ignacio Conget, Jorge Bondia, Josep Vehí

**Affiliations:** 1 Institut d’Informàtica i Aplicacions, Universitat de Girona, 17003 Girona, Spain; lyviar@utfpr.edu.br (L.B.); abertachi@utfpr.edu.br (A.B.); 2 Federal University of Technology—Paraná (UTFPR), Guarapuava 85053-525, Brazil; 3 Diabetes Unit, Endocrinology and Nutrition Department, Hospital Clínic Universitari, IDIBAPS (Institut d’investigacions Biomèdiques August Pi i Sunyer), 08036 Barcelona, Spain; cmquiros@clinic.cat (C.Q.); gimenez@clinic.cat (M.G.); iconget@clinic.cat (I.C.); 4 Centro de Investigación Biomédica en Red de Diabetes y Enfermedades Metabólicas Asociadas (CIBERDEM), 28029 Madrid, Spain; jbondia@isa.upv.es; 5 Instituto Universitario de Automática e Informática Industrial, Universitat Politècnica de València, 46022 Valencia, Spain

**Keywords:** continuous glucose monitoring, accuracy, exercise, physical activity, type 1 diabetes

## Abstract

Continuous glucose monitoring (CGM) plays an important role in treatment decisions for patients with type 1 diabetes under conventional or closed-loop therapy. Physical activity represents a great challenge for diabetes management as well as for CGM systems. In this work, the accuracy of CGM in the context of exercise is addressed. Six adults performed aerobic and anaerobic exercise sessions and used two Medtronic Paradigm Enlite-2 sensors under closed-loop therapy. CGM readings were compared with plasma glucose during different periods: one hour before exercise, during exercise, and four hours after the end of exercise. In aerobic sessions, the median absolute relative difference (MARD) increased from 9.5% before the beginning of exercise to 16.5% during exercise (*p* < 0.001), and then decreased to 9.3% in the first hour after the end of exercise (*p* < 0.001). For the anaerobic sessions, the MARD before exercise was 15.5% and increased without statistical significance to 16.8% during exercise realisation (*p* = 0.993), and then decreased to 12.7% in the first hour after the cessation of anaerobic activities (*p* = 0.095). Results indicate that CGM might present lower accuracy during aerobic exercise, but return to regular operation a few hours after exercise cessation. No significant impact for anaerobic exercise was found.

## 1. Introduction

Continuous glucose monitoring (CGM) is associated with improvement in glycaemic control in patients with type 1 diabetes (T1D), reducing glycated haemoglobin (HBA_1C_) percentage without increasing the occurrence of hypoglycaemic episodes [1,2,3]. Using a minimally invasive sensor inserted in the subcutaneous tissue, patients can follow their readings in real-time, allowing them to make changes in their therapy to improve glycaemic variability and metabolic control safely. Additionally, CGM data allows physicians to visualise individualised glycaemic traces along consecutive days, and then improve patients’ insulin treatment.

Although regular physical activity (PA) is recommended for patients with T1D to improve overall health conditions [4], diabetes management in front of exercise is not a trivial task and monitoring glucose levels before, during, and after physical activity is fundamental to maintaining plasma glucose (PG) levels in euglycaemic ranges during and after exercise [5]. Artificial pancreas systems (also referred to as closed-loop systems) rely on CGM readings to automatically calculate at every sampling time the necessary insulin dose to keep patients’ PG levels in safe ranges [6]. In 2017, the first artificial pancreas system hit the market in United States, although still requiring patient intervention at mealtimes [7]. 

PA has been identified as one of the major challenges facing artificial pancreas systems [8]. PA is also a hurdle for CGM accuracy, due to changes in subcutaneous tissue circulation, variations in oxygen concentration of blood, increase in body temperature, mechanical forces where the sensor is placed, and rapid glucose changes in glucose concentrations caused by exercise [9]. Nevertheless, if CGM accuracy is poor, the closed-loop performance may be deteriorated [10] and thus increase the intrinsic risk of hypo- and hyperglycaemia caused by PA. Several studies have reported the impact of PA in the accuracy of current-generation CGM systems [9,11,12,13]. To the best of our knowledge, no study has yet investigated the “dynamic” behaviour of the accuracy of CGM devices in face of exercise, as measured at time intervals before, during, and after exercise sessions. Thus, the aim of this study is to evaluate the accuracy of CGM before, during, and after aerobic and anaerobic exercise sessions performed by patients with T1D. In addition, the post-exercise period was split into four consecutive 1-h periods, allowing visualisation of the accuracy of CGM devices along the recovery period.

## 2. Materials and Methods

### 2.1. Study Population

Six patients with T1D were enrolled at the Clinic University Hospital of Barcelona. The protocol was approved by the Ethics Committee of the hospital. Subjects were eligible to participate if they were between 18 and 60 years old, with a body mass index (BMI) between 18 and 30 kg/m^2^, HbA_1C_ between 6.0% and 8.5%, and were on continuous subcutaneous insulin infusion (CSII) for at least six months. Patients using any experimental drug or device in the past 30 days were excluded. Patients with progressive fatal diseases, hypoglycaemia unawareness, a history of drug or alcohol abuse, impaired hepatic, neurological, endocrine, or other systematic diseases apart from T1D, and/or pregnant women were also excluded. Table 1 shows the demographic characteristics of the study population.

### 2.2. Study Procedures

The study was a longitudinal, prospective, interventional study with a primary objective of the analysis of the limits of performance of a closed-loop controller when challenged by PA and, as a secondary objective, the analysis of the impact of exercise in continuous glucose monitoring accuracy. Each subject underwent three aerobic and three anaerobic exercise tests, completing six experiments in about nine weeks. CSII was carried out with the Paradigm Veo^®^ insulin pump and the day before the test, the patient inserted two Enlite-2 sensors^®^, both by Medtronic Minimed (Northridge, CA, USA), subcutaneously at home. Sensors were inserted in the abdomen. Patients arrived at the research unit at 8:00, after a standardised breakfast at home. PG samples were measured every 15 min using a YSI 2300 Stat Plus Glucose Analyser (YSI Inc., Yellow Springs, OH, USA). In cases where PG readings were below 80 mg/dL, measurements were performed every five minutes. To ensure comparable metabolic conditions between studies, subjects received intravenous infusion of regular human insulin to maintain plasma glucose at 150 mg/dL until the beginning of studies at 11:00, according to a feedback insulin infusion method. Just before 11:00 patients ingested 23 grams of carbohydrates (15 g as gel—*Diabalance gel de glucosa acción rápida* (Esteve, Barcelona, Spain) and 8 g as an isotonic drink—100 mL of Aquarius (The CocaCola Company, Atlanta, GA, USA)). At 10:45, the closed loop system started and at 11:00 the exercise protocol started. Two exercise protocols were considered: aerobic and anaerobic. The trials with aerobic exercises consisted of three series of 15 min of cycle-ergometer at 60% of the individual maximal O_2_ consumption (VO_2_max), with 5 min of rest between sets. In the trials with anaerobic exercises, the patient performed five sets of eight repetitions of four different exercises at 70% of the maximum capacity with 90 s of rest between sets of weightlifting.

### 2.3. Data and Statistical Analysis

Median absolute relative difference (MARD) was analysed in six different periods (P0 to P5, all lasting one hour). P0 is the one-hour period before the exercise session, P1 is during the exercise, and P2 to P5 are the periods after the exercise session (comprising 4 h post-exercise). In total, 36 exercise sessions were performed, in which two sensors were used per patient. From these 36 sessions, seven complete sessions and two sensors were discarded due to a malfunction of YSI or CGM unit. The total number of sensors analysed for the aerobic and anaerobic sessions was 31 and 25, respectively.

CGM data were recorded every five minutes. In order to align CGM and PG data, CGM data were linearly interpolated and rounded to one sample per minute. Missing data were not interpolated. MARD was computed per sensor, considering all periods (P0 to P5), in order to check for outliers. After that, the detected outlier sensors were also discarded, giving rise to a final number of sensors analysed for the aerobic and anaerobic sessions of 28 and 22, respectively.

Sensor accuracy is presented for each period and compared between them. In addition, the mean PG of each period is also presented, considering that accuracy may be deteriorated in the hypoglycaemic range [10,14]. Descriptive statistics of the MARD results are given as medians (interquartile range (IQR)). A comparison of medians from MARDs in different periods was performed with the two-sided Wilcoxon rank sum test, considering a significance level of 0.05. Paired PG reference and CGM readings for both aerobic and anaerobic exercises are given using the Clarke error grid analysis [15].

## 3. Results

### 3.1. Aerobic Exercise

**Figure 1:**
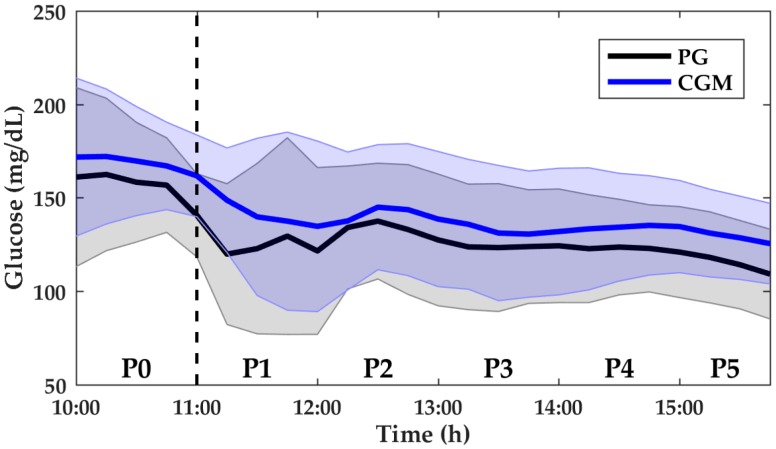
Plasma glucose (PG) and continuous glucose monitoring (CGM) measurements from P0 to P5 for aerobic sessions. Data shown are mean ± standard deviation (SD). PG is denoted by the black solid line and black-shaded area. CGM is denoted by the blue solid line and blue-shaded area. The vertical dashed line indicates the start of aerobic exercise sessions.

Figure 1 illustrates CGM and PG measurements during the entire period of accuracy analysis. During this period, the mean CGM was 142.53 ± 14.64 mg/dL and the mean PG was 130.53 ± 14.98 mg/dL. Table 2 presents the values of median PG and accuracy for all the periods P0–P5 and the amount “*n*” of paired CGM and PG samples in each period for MARD computation. Table 3 depicts the *p*-value between each period.

**Figure 2:**
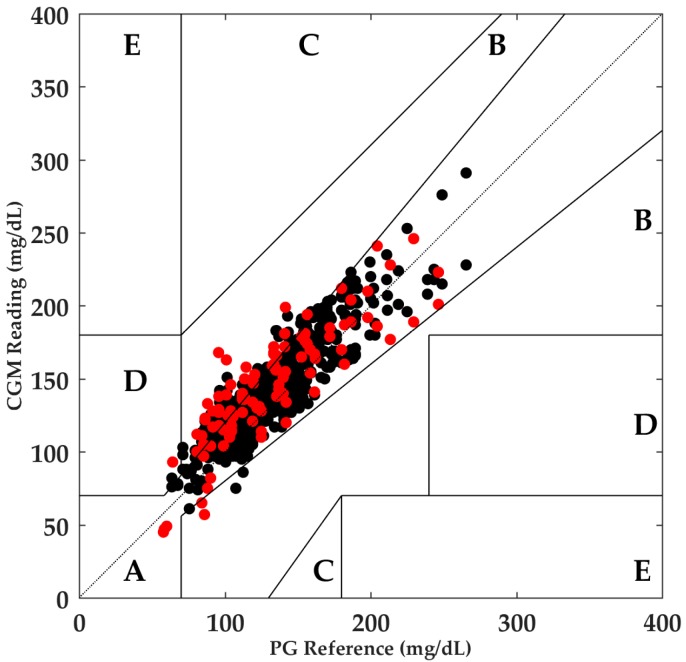
Clarke error grid analysis of CGM and PG values for aerobic exercise. Zone A: 81.6%, Zone B: 18.1%, Zone C: 0.0%, Zone D: 0.3%, Zone E: 0.0%. Red points illustrate the readings during P1. CGM, continuous glucose monitor; PG, plasma glucose.

The results showed a degradation of accuracy caused by the onset of aerobic exercise. Additionally, the MARD obtained during P1 was significantly different when compared with the remaining periods, evidencing the effects caused by aerobic exercise on CGM accuracy. The Clarke error grid analysis showed that 99.7% of the points during all the periods were in Zones A and B, considered clinically acceptable. During the exercise period (P1), the red points illustrated in Figure 2 were distributed as follows: Zone A: 63.9%, Zone B: 35.2%, Zone C: 0.0%, Zone D: 0.9%, and Zone E: 0.0%.

### 3.2. Anaerobic Exercise

**Figure 3:**
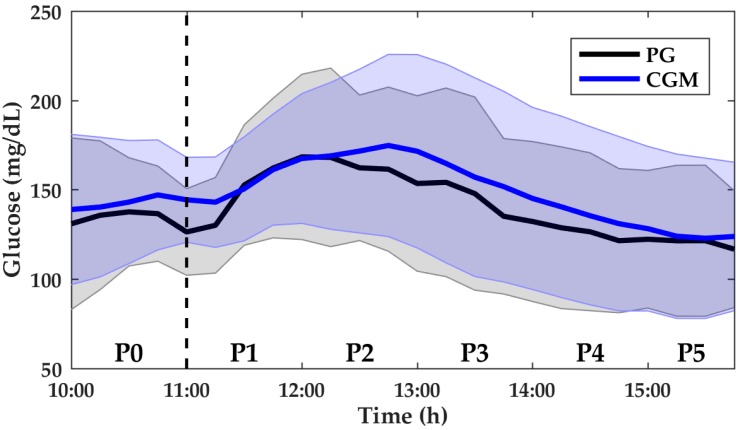
PG and CGM measurements from P0 to P5 for anaerobic sessions. Data shown are mean ± SD. PG is denoted by the black solid line and black-shaded area. CGM is denoted by the blue solid line and blue-shaded area. The vertical dashed line indicates the start of the anaerobic exercise sessions. PG, plasma glucose; CGM, continuous glucose monitor; SD, standard deviation.

In the anaerobic exercise sessions, the mean CGM was 147.82 ± 16.29 mg/dL and the mean PG was 139.75 ± 16.58 mg/dL. Figure 3 shows CGM and PG over the whole period. Table 4 presents the MARD for each period as well as the median PG values. Table 5 depicts the *p*-value between each period.

**Figure 4:**
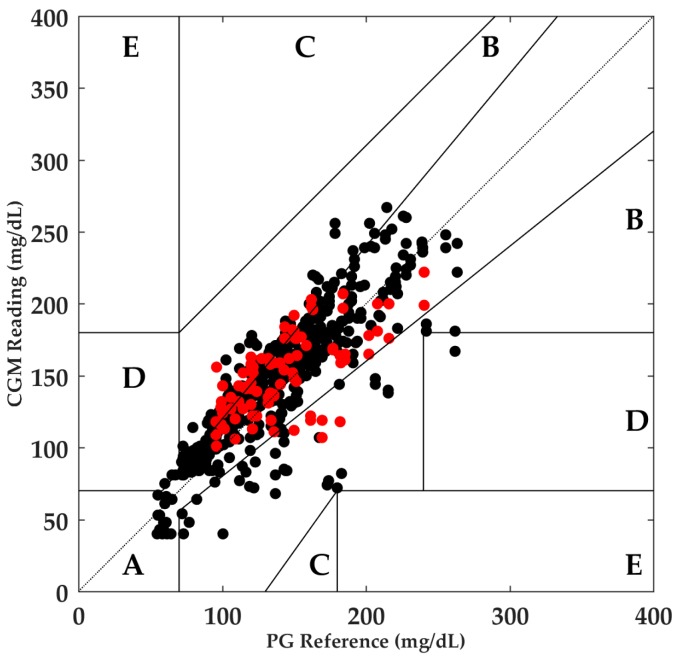
Clarke error grid analysis of CGM and PG values for anaerobic exercise. Zone A: 70.5%, Zone B: 28.9%, Zone C: 0.0%, Zone D: 0.6%, and Zone E: 0.0%. Red points illustrate the readings during P1. CGM, continuous glucose monitor; PG, plasma glucose.

Different from what was observed in the aerobic results, the onset of anaerobic exercise did not have an influence on MARD. Despite the fact that the MARD slightly increased during P1 compared with P0 (16.8% vs. 15.5%), this increase was not statistically significant. In the following period, a greater drop in MARD was observed; however, no statistical significance was observed. Considering the entire period, 99.4% of the paired points were distributed in Zones A and B in the Clarke error grid. In the exercise period (P1), the red points illustrated in Figure 4 were distributed as follow: 61.6% were in Zone A and the remaining 38.4% were in Zone B.

## 4. Discussion and Conclusions

An unexpected MARD difference in the period of P0 between aerobic and anaerobic exercise was found (MARD = 9.5% vs. 15.5%, *p* < 0.01), probably due to random factors. However, this fact did not affect the purpose of the analysis, which was the characterisation of the MARD degradation, if any, in both types of exercise. Indeed, such degradation was found in the case of aerobic exercise, with a 73% increment in MARD (from 9.5% to 16.5%, *p* < 0.001) during the time the exercise was performed. This might be due to O_2_ changes, microcirculation perturbation, or movement around or within the insertion area and sensor during the cycling exercise. However, after exercise cessation, the MARD value returned to values comparable to the baseline (see Table 3). A non-statistically significant increase was observed in the MARD during the anaerobic sessions (from 15.5% to 16.8%, *p* = 0.993). However, such small differences may have been affected by the elevated MARD during P0 as well as by the small size of the cohort. These facts hinder any definitive conclusion about the behaviour of the MARD during this kind of exercise. The MARD for P5 (the fourth hour after the end of exercise sessions) was similar to that found in Reference [10], which reports an overall mean absolute relative difference for the same sensor during the postprandial period of 12.0%. Additionally, the results for P5 are similar to the MARD presented in Reference [11] (11.95%), which is result of the analysis of the accuracy of the first- and second-generation Enlite sensors during a rest period. These results indicate that the sensors, even if affected by exercise, might be able to return to their regular operation a few hours after exercise cessation.

Additionally, a trend in the overestimation of CGM can be observed during all of the periods analysed for aerobic trials. In the anaerobic trials, no such trend was observed. Although CGM overestimated PG in three different periods (P0, P3, P4), this trend did not occur in the remaining periods.

The impact of physical activity on CGM accuracy was also observed by other studies [9,11,12,13]. In Reference [9], CGM accuracy was also affected by aerobic exercise in pregnant women with T1D that undertook 55 min of moderate walking on a treadmill. During the exercise period, the MARD was greater than that during sedentary period (18.4% vs. 11.5%, *p* < 0.001). In Reference [11], the authors showed that two different CGM systems, Dexcom G4 Platinum (Dexcom, San Diego, CA, USA) and Medtronic Paradigm Veo Enlite systems, presented lower performance during continuous and interval exercise sessions when compared with a rest period. Furthermore, they observed a trend in the Medtronic Paradigm Veo Enlite system to obtain worse accuracy during continuous exercise than interval exercise. Although in our work patients only used the second-generation Enlite sensors (in Reference [11] the first-generation sensors were used in 55% of the experiments), continuous aerobic exercise had a more negative impact in CGM accuracy. In Reference [12], no statistically significant difference was observed in CGM accuracy during continuous and high-intensity interval exercise (HIIE) in young adults with T1D, and the MARDs achieved in this study were similar to the MARD obtained in another inpatient study [14] that considered the same sensor, but without exercise. In Reference [13], the authors detected a trend caused by HIIE in the overestimation of CGM for different levels of intensity. However, for continuous exercise, CGM only overestimated the reference levels for low-intensity activity.

An important limitation in our study is the small size of the cohort. Only six patients were enrolled in this study, with a single female. However, three sessions per exercise type were performed by each patient, using two simultaneous sensors in each session. Another limitation was that there were no variations in the level of exercise intensity for any type of exercise. This impeded the assessment of the effects of exercise intensity on CGM performance. The difference observed in the MARD before the onset of exercise (P0) did not allow a direct comparison between each type of exercise.

CGM technology helps patient to improve overall glycaemic control. The focus of the present paper was to analyse CGM accuracy in different exercise conditions. We concluded that the accuracy of the second-generation Medtronic Enlite sensor is affected by aerobic exercise. Further improvement of CGM technology may be needed in order to reassure robustness and safety during PA.

## Figures and Tables

**Table 1:** Characteristics of the patients.

Number of Patients (Females)	6 (1)
Age (years) *	36.7 ± 8.9
HbA_1C_ (%) *	7.9 ± 0.5
Body mass index (kg/m^2^) *	24.6 ± 1.0
Time with T1D (years) *	25.2 ± 12.7
Time with pump (years) *	4.8 ± 1.7

* Data expressed as mean ± standard deviation. T1D, type 1 diabetes.

**Table 2:** Values of PG and accuracy of CGM for aerobic exercise sessions.

	P0	P1	P2	P3	P4	P5
PG (mg/dL)	155.0 (135.3–174.5)	120.5 (99.5–149.9)	124.0 (106.8–155.8)	114.5 (101.3–146.3)	118.3 (104.5–143.8)	111.5 (101.0–129.8)
MARD (%)	9.5 (4.7–13.9)	16.5 (7.6–23.5)	9.3 (5.4–16.3)	11.6 (6.5–17.5)	11.3 (6.2–16.0)	12.9 (4.7–18.8)
*n*	112	108	108	108	108	108

Data are expressed as the median (interquartile range). PG, plasma glucose; CGM, continuous glucose monitor; MARD, median absolute relative difference.

**Table 3:** *p*-Value between each period analysed for aerobic exercise.

	P0	P1	P2	P3	P4	P5
**P0**	–	<0.001	0.986	0.177	0.281	0.060
**P1**	<0.001	–	<0.001	<0.01	<0.001	<0.05
**P2**	0.986	<0.001	–	0.189	0.346	0.075
**P3**	0.177	<0.01	0.189	–	0.683	0.452
**P4**	0.281	<0.001	0.346	0.683	–	0.241
**P5**	0.060	<0.05	0.075	0.452	0.241	–

**Table 4:** Values of PG and accuracy of CGM for anaerobic exercise sessions.

	P0	P1	P2	P3	P4	P5
PG (mg/dL)	124.5 (110.8–155.8)	138.0 (118.3–160.0)	157.5 (139.5–195.0)	149.3 (108.5–179.8)	138.5 (84.5–159.0)	129.8 (88.3–150.8)
MARD (%)	15.5 (6.5–26.4)	16.8 (7.9–24.5)	12.7 (4.9–20.3)	14.3 (4.8–26.5)	14.3 (7.9–19.7)	12.3 (5.7–18.8)
*n*	*n* = 76	*n* = 86	*n* = 88	*n* = 88	*n* = 89	*n* = 88

Data are expressed as median (interquartile range). PG, plasma glucose; CGM, continuous glucose monitor; MARD, median absolute relative difference.

**Table 5:** *p*-Value between each period analysed for anaerobic exercise.

	P0	P1	P2	P3	P4	P5
**P0**	–	0.993	0.095	0.709	0.287	<0.05
**P1**	0.993	–	0.063	0.798	0.171	<0.05
**P2**	0.095	0.063	–	0.188	0.444	0.691
**P3**	0.709	0.798	0.188	–	0.458	0.076
**P4**	0.287	0.171	0.444	0.458	–	0.223
**P5**	<0.05	<0.05	0.691	0.076	0.223	–
